# Effect of Spatial Separation of Pigs on Spread of *Streptococcus suis* Serotype 9

**DOI:** 10.1371/journal.pone.0061339

**Published:** 2013-04-10

**Authors:** Niels Dekker, Annemarie Bouma, Ineke Daemen, Don Klinkenberg, Leo van Leengoed, Jaap A. Wagenaar, Arjan Stegeman

**Affiliations:** 1 Faculty of Veterinary Medicine, Department of Farm Animal Health, Utrecht University, Utrecht, The Netherlands; 2 Faculty of Veterinary Medicine, Department of Infectious Diseases and Immunology, Utrecht University, Utrecht, The Netherlands; Cornell University, United States of Amrrica

## Abstract

The spread of an infectious agent in a population can be reduced by interfering in the infectiousness or susceptibility of individuals, and/or in their contact structure. The aim of this study was to quantify the effect of prevention of direct contact between infectious and susceptible pigs on the transmission of *Streptococcus suis* (*S. suis)*. In three replicate experiments, *S. suis*-free pigs were housed in boxes either in pairs (25 pairs) or alone (15 pigs). The distance between the boxes was ±1 m. At 7 weeks of age, one pig of each pair was inoculated intranasally with *S. suis* serotype 9; the other pigs were exposed to *S. suis* by either direct (pairs) or indirect contact (individually housed pigs). Tonsillar brush and saliva swab samples from all pigs were collected regularly for 4 weeks post inoculation to monitor colonization with *S. suis*. All inoculated pigs became infected, and their pen mates became colonized within 2 days. Thirteen indirectly exposed pigs became positive within 7–25 days after exposure. The rate of direct transmission *β_dir_* was estimated to be 3.58 per pig per day (95% CI: 2.29–5.60). The rate of indirect transmission increased in time, depending on the cumulative number of days pigs tested positive for the presence of *S. suis.* The estimate *β’_ind_* was 0.001 (95% CI: 0.0006–0.0017) new infections per pig per day for each day that an infected pig was tested positive for *S. suis*. We conclude that prevention of direct contact reduces the rate at which susceptible pigs become colonized. Simulation studies using these parameters showed, however, that such intervention measure would not limit *S. suis* serotype 9 spread in a commercial pig farm to a relevant extent, implying that spatial separation of groups op pigs within a compartment would not be effective on a farm.

## Introduction

Infectious diseases are a large problem in animal husbandry and various control measures are implemented to reduce their impact, either by blocking transmission of the infection or by minimizing disease upon infection. Transmission of an infection depends on the infectiousness of infected individuals, the susceptibility of uninfected ones, and their mutual interaction [1]–[4]. Direct contact between infected and susceptible animals is considered to be the most important risk factor for spread of infections [5]–[8]. Consequently, interference in the contact structure between infected and susceptible animals, either by reducing the frequency or the intensity of the contact, might contribute to reduction of transmission of infections within a farm [2], [5], [6].

Separation of animals by adjustments of the housing or management systems has been applied on dairy and sheep farms to prevent transmission of pathogens from dams to offspring [5], [9]. Implementation of this measure is also considered for pig farms to improve the health status [10]–[13]. Measures to reduce contacts between pigs, however, do not fit well in current pig management practices for various reasons. One is that it is current management practice to mix pigs to create homogeneous groups; another reason is that individual housing is not acceptable for welfare reasons. Therefore, before considering measures to reduce infection transmission by separation of pigs or groups of pigs, it should be demonstrated that such measures have a substantial effect.

Evaluation of such separation measures can be performed at different scales, e.g. at region scale, with herds as units, at herd scale, with compartments as units, or at compartment scale, with individual pigs as units. The dynamics of spread of a pathogen among individuals within a compartment highly determines the dynamics at a higher scale e.g. between compartments or farms. Consequently, the effectiveness of a measure with respect to reducing the spread of a pathogen at a higher scale depends on the effect at a lower scale [14]. As a first step, we therefore focus on the lowest scale, i.e. individual pigs in a compartment.

One of the infectious agents on pig farms is *Streptococcus suis* (*S. suis*), which may cause meningitis, arthritis or septicaemia in piglets. The prevalence of infected pigs differs between farms, and varies with age [15]–[26]; mortality up to 20% has been reported [27], [28].Several serotypes of *S. suis* are circulating, of which serotypes 2 and 9 are most often isolated from clinical cases [28]–[33]. Disease caused by *S. suis* is a major determinant for abundant use of antibiotics in pig farming [34]. Prudent use of antimicrobial therapy is propagated and therefore alternative control measures should be seriously considered.

Many routes have been suggested for pig-to-pig transmission of *S. suis*. As *S. suis* is frequently isolated from the pigs upper respiratory tract, the direct oro-nasal route is generally assumed an important one [27], [28]. Other, indirect routes are also possible (e.g. airborne) [35].

The aim of our study was to determine whether the spread of *S. suis* within a compartment could be reduced by prevention of direct contact between pigs. We quantified the transmission rate of *S. suis* serotype 9 in an experimental set up between pigs housed in pairs and between pigs housed in pens placed at a distance of approximately 1 m. Such a design has been considered as robust in quantifying transmission [36], [37], and has been used for several other bacterial pathogens [38]–[40]. The parameter estimates derived from our experiments were used in a simulation model to study the effect of separation of pigs in a hypothetical conventional farm. Our experimental results show that the transmission rate is influenced by contact structure between pigs, but the simulation showed that separation most likely does not restrict spread of *S. suis* in a conventional stable compartment.

## Materials and Methods

### Ethics Statement

The experiments were approved by the Animal Care and Ethics Committee of Utrecht University, in accordance with the Dutch law on experimental animals (approval number DEC 2008.II.08.072). To reduce the number of animals, we carried out experiments to study the effect of vaccination on transmission of *S. suis* as well. The results of that research objective have been published elsewhere [41].

### Inoculum

*S. suis* serotype 9 strain 7997 was used as inoculum (provided by H. Smith, Central Veterinary Institute, Wageningen UR, Lelystad, The Netherlands). The strain was isolated from a clinical case in a commercial farm in The Netherlands. The strain contains genes encoding for several (putative) virulence factors, like suilysine (SLY) and variant (higher molecule weight) muramidase released protein (MRP*) [42], and belongs to the clonal complex (CC) that includes the vast majority of invasive *S. suis* serotype 9 strains found in the Netherlands, i.e. CC16 [43]. After overnight culture from a −80°C stock on agar plates at 37°C and 5% CO_2_, one colony was suspended in 10 mL Todd-Hewitt broth (TH) (BioTrading, The Netherlands), and incubated for 3–4 h at 37°C until an optical density of 0.5–0.6 at 600 nm. After overnight storage at 4°C this suspension was diluted tenfold in TH and cultured for 2 h at 37°C resulting in an optical density of 0.5–0.6 at 600 nm. Ten mL of this suspension was washed twice and suspended in 10 mL physiologic saline solution. The bacterial concentration of the final suspension was 2–3×10^8^ colony forming units (CFU) per mL.

### Pigs

We used Landrace×Yorkshire pigs from sows housed at the animal facilities of the department. Piglets were caesarean derived and colostrum deprived. The first 4 weeks of life the piglets were housed in isolators, and thereafter in ground floor pens (12–15 pigs/pen). The pigs were fed with milk replacers during the first 4 weeks, and then with gamma-irradiated pelleted concentrates (Sloten B.V., The Netherlands; Trouw Nutrition Nederland B.V., The Netherlands). The feed contained *Enterococcus faecium* (DSM 7134), *Bacillus licheniformis* (DSM 5749) and *Bacillus subtilis* (DSM 5750).

### Experimental design

**Figure 1:**
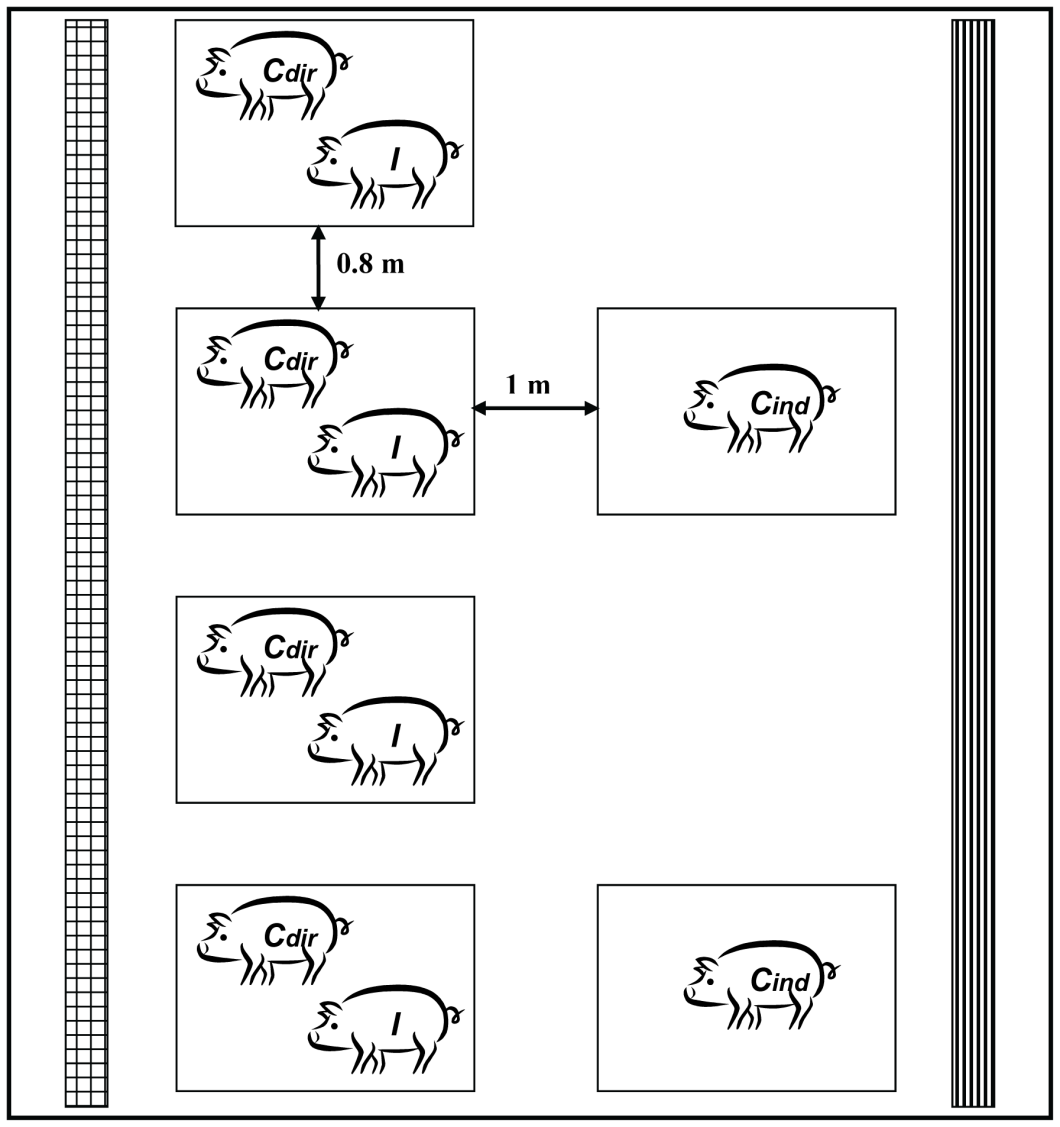
Design of stable compartment in animal experiments. Experimental design for evaluation of the effect of prevention of direct contact by spatial separation on transmission of *S. suis* serotype 9 among pigs. Inoculated pigs (*I*) were intranasally infected with *S. suis* serotype 9 two days before placing in these boxes. Stable compartments contained 2 to 4 boxes with pair-wise housed pigs (*C_dir_*+*I*) and 1 to 3 boxes with pigs housed individually (*C_ind_*). *C_dir_* pigs had direct contact with *I* pigs; *C_ind_* pigs were only indirectly exposed to *S. suis*. The air inlet in the compartment is marked with 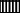; the air outlet with 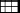.

Three replicate experiments were conducted sequentially (for details: see Table 1 and Figure 1). Pigs were housed either in pairs or alone in boxes. Pair-wise housed pigs were used to measure direct transmission, the pigs housed alone to determine indirect transmission. The distance between boxes was 80–100 cm. This distance was sufficient to prevent direct contact between pigs in different pens, and might be applicable between groups in commercial farms. The boxes had multiplex walls (height: 80 cm), iron grid floor with rubber lying area, with a total area of 1.2 m^2^ per box.

**Table 1:** Design of animal experiments to evaluate the effect of spatial separation on *S. suis* serotype 9 transmission.

	stable compartment		individually housed	pair-wise housed	
experiment		N pigs	N indirect contacts	N direct contacts	N inoculated pigs
I	A	7	1	3	3
	B	5	1	2	2
					
II	A	10	2	4	4
	B	10	2	4	4
	C	6	2	2	2
					
III	A	10	2	4	4
	B	10	2	4	4
	C	7	3	2	2

At the age of 7 weeks, one pig in each pair was inoculated intranasally with 1×10^9^ CFU *S. suis* in 5 mL saline while sedated. Inoculation was performed in another stable compartment to prevent infection of contacts due to spread of the inoculum. Two days later the inoculated pigs were placed back into their original boxes. The pen mates were considered to be directly exposed, the pigs housed alone to be indirectly exposed. In total 25 inoculated (referred to as *I*), 25 direct contact pigs (referred to as *C_dir_*) and 15 individually housed, indirect contact pigs (referred to as *C_ind_*) were used. Compartments contained 2–4 boxes with pairs and 1–3 boxes with individually housed pigs, and per experiment 2–3 separate compartments were used (Table 1 and Figure 1).

Rectal temperatures and clinical observations were recorded daily. Neurologic signs and signs caused by lesions of the locomotor apparatus were scored separately. Pigs that showed severe clinical signs were euthanized. The remaining pigs were euthanized at 26–28 days post inoculation (DPI).

Biosecurity protocols were applied to prevent contact infections by animal handling. Animal handlers wore sterile gloves, boots and coveralls which were changed before handling between each box. *C_ind_* pigs were sampled first, and *C_dir_* pigs were sampled before *I* pigs.

To study the effect of vaccination on transmission of *S. suis* 13 of the 25 pairs of pigs (see Table S1 for details) were vaccinated at 4 and 7 weeks of age with a formalin inactivated whole bacterin vaccine, containing the same *S. suis* strain as used for inoculation (homologous vaccination). The results showed that both level of *S. suis* colonization and within-pen transmission were not affected by vaccination [36].

### Sampling

Saliva and tonsil brushing samples were taken at 5 days and 3 h before inoculation, and 2–7, 9, 12, 15, 19, 22 DPI, and at the end of the experiment. We used a sterile steel wedge to open the mouth; saliva was sampled by turning round a swab (Cultiplast®) under the tongue for 5 s. Both palatine tonsillar areas were brushed for 3 s each with a sterile toothbrush. The brush and swab heads were put in separate sterile tubes containing 10 mL saline solution and transported to the laboratory. All pigs were necropsied and macroscopically affected organs were, if present, sampled for bacteriological examination. Palatine tonsillar tissue was collected.

### Laboratory tests

Quantitative bacteriological examination was performed on all samples. Serial dilutions of swab or tonsil brushing samples (10^0^–10^−4^) were plated on selective agar plates, containing Columbia agar, 6% sheep blood, 0.2 µg/mL crystal violet and colistin/oxolinic acid (BioTrading, The Netherlands) [41], [44]. After incubation for 18–24 h at 37°C and 5% CO_2_, plates with 10–200 colonies were selected. Suspected *S. suis* colonies were counted and per plate two *S. suis* suspected colonies were subcultured and tested for amylase activity [45]. Isolates that showed amylase activity were stored at −20°C in 0.5 mL TE buffer pH 7.5 (10 mM) until further processing. DNA-isolation of these isolates was performed with InstaGene™ Matrix (Biorad, The Netherlands), according to the manufacturers instructions. A real time polymerase chain reaction (RT-PCR) on the *cps9H* gene [41], [46] was performed on suspected isolates from the first two and the last saliva and tonsil samples in which amylase positive colonies were detected. All colonies that were visually classified as *S. suis* tested amylase-positive, and were all positive in the *cps9H*-PCR. Tonsil tissue samples that were obtained at necropsy were submerged in boiling water (5 s), crunched in a Stomacher® macerator (Laméris, The Netherlands) for 10 min, and diluted, plated and confirmed as described above for samples, except for the dilutions that were plated (10^−1^ to 10^−5^).

### Statistical analysis of culture data and clinical data

Mean *S. suis* ^10^logCFU levels of the first 3 samples taken after onset of colonization in individual pigs were compared among the 3 groups (*C_dir_*, *C_ind_* and *I*) using a Kruskal-Wallis one-way ANOVA. The same procedure was performed for mean levels over the whole period after pigs tested positive, and for *S. suis* counts (in ^10^logCFU/g tissue) in tonsillar tissue. For samples where no *S. suis* was detected, the detection limit of the test procedure (i.e. 2×10^3^ CFU/sample) was used for the calculations. It did not affect the results if we had used a much lower value, i.e. 2 CFU/sample, instead for analysis.

Clinical signs were compared between the groups with a Kruskal-Wallis test applied on the percentage of days a pig showed signs. If the result of the Kruskal-Wallis test was significant, the Mann-Whitney U test was performed to determine differences between groups. The Bonferroni approach was used to correct for multiple comparisons. Differences in outcome variables were considered significant if *P*<0.05. Analysis of culture and clinical data was performed in SPSS version 16.0.2.

### Statistical analysis of transmission

To analyze transmission of *S. suis*, a stochastic SI (susceptible-infectious) model was applied. A pig was considered to be infected if at least one sample tested positive. The transmission rates *β_dir_* and *β_ind_* represent the rates at which infectious animals transmit the infection to other animals within and among pens, respectively. The parameter *β_ind_* includes the process of *S. suis* transmission without direct contact between hosts, and thus exclusively via the environment. The parameter *β_dir_* includes both transmission via direct contact and transmission via the environment, as these two routes cannot be distinguished in a pair wise setting.

According to the standard SI model, susceptible pigs become infectious at rate *β_dir_*_ *_ *I*_w_ (or *β_ind_*_ *_ *I_b_*) in which *I*_w_ and *I_b_* are the numbers of infectious animals present within the same pen, and in the whole compartment, respectively. The standard model assumes that susceptible animals are infected at a constant rate if *I_w_* (or *I_b_*) is constant, which was more or less constant during our experiment. For within-pen transmission the data allowed the standard model to be used. For between-pen transmission, however, we did not see new infections in *C_ind_* until one week after exposure to inoculated pigs, and counted 13 cases in the two weeks thereafter. This implies that for between-pen transmission the assumption of constant infection rate per unit of time was not met, and the standard SI model could not be used for analysis. For between-pen transmission, we therefore fitted an alternative model in which susceptible animals are infected at rate *β’_ind_* * *ΣI*, in which *ΣI* is the cumulative number of shedding days of all infectious animals in that stable compartment (at time *t*). In this model a shedding day was defined as a day that *S. suis* was found in the tonsil and/or nasal swab sample taken at that day. This model reflects a gradual build-up of infectivity in the environment. The alternative transmission rate *β’_ind_* (unit: 1/day^2^) is now interpreted as the number of new infections an animal will cause *each day* in a susceptible population, *for each day* that it has already been infectious. In the analysis it was assumed that from the moment that pigs died or were euthanized, they did not add any infectivity to the cumulative infectivity anymore. The infectivity they had added before death was still included in the total infectivity in that compartment for the remaining experimental period.

Additionally, as the bacterial load in infected pigs might influence the transmission rate, models were tested which included cumulative colonization or shedding levels in tonsillar brushing or saliva samples instead of shedding days. The ‘cumulative colonization or shedding level’ at a particular day was calculated by adding the CFU/sample up to that day, using interpolation for all days on which no sample was taken. We used Akaike's Information Criterion (AIC) to test if this alternative model fitted the data better than the original model [47]. The transmission rate calculations were performed in statistical program R version 2.13.0.

As mentioned, the experiment was designed to serve a dual purpose to reduce the number of experimental animals. As vaccinated and non-vaccinated pigs were distributed over all but one compartments, it was not possible to distinguish between infectivity of vaccinated and unvaccinated pairs. In addition, the bacterial load in swab or brushing samples did not differ significantly between vaccinated and unvaccinated pigs. It was therefore assumed that all colonized pigs contributed equally to transmission of *S. suis*. Therefore, vaccination was not included as explanatory variable in the statistical analysis to determine the effect of spatial separation. Power analysis was based on the research question about vaccination [41].

### Simulation study

The effect of separation of groups of pigs on the spread of *S. suis* in a conventional farm compartment was studied using a simulation model. A situation with separated litters (scenario A) was compared with one of mixing litters (scenario B). In scenario A, the compartment contained 10 pens each consisting of 10 pigs. In scenario B, 10 litters of 10 pigs were mixed to form two groups of 50 pigs housed in two pens. The model was independent of distances between pens. Simulations were started with one infected pig. The estimate for *β’_ind_* was divided by two to compensate for the in comparison to a compartment extra space needed to house 100 pigs. Per situation 10.000 iterations were performed. Simulation was done with statistical program R version 2.13.0.

## Results

### Colonization

**Figure 2:**
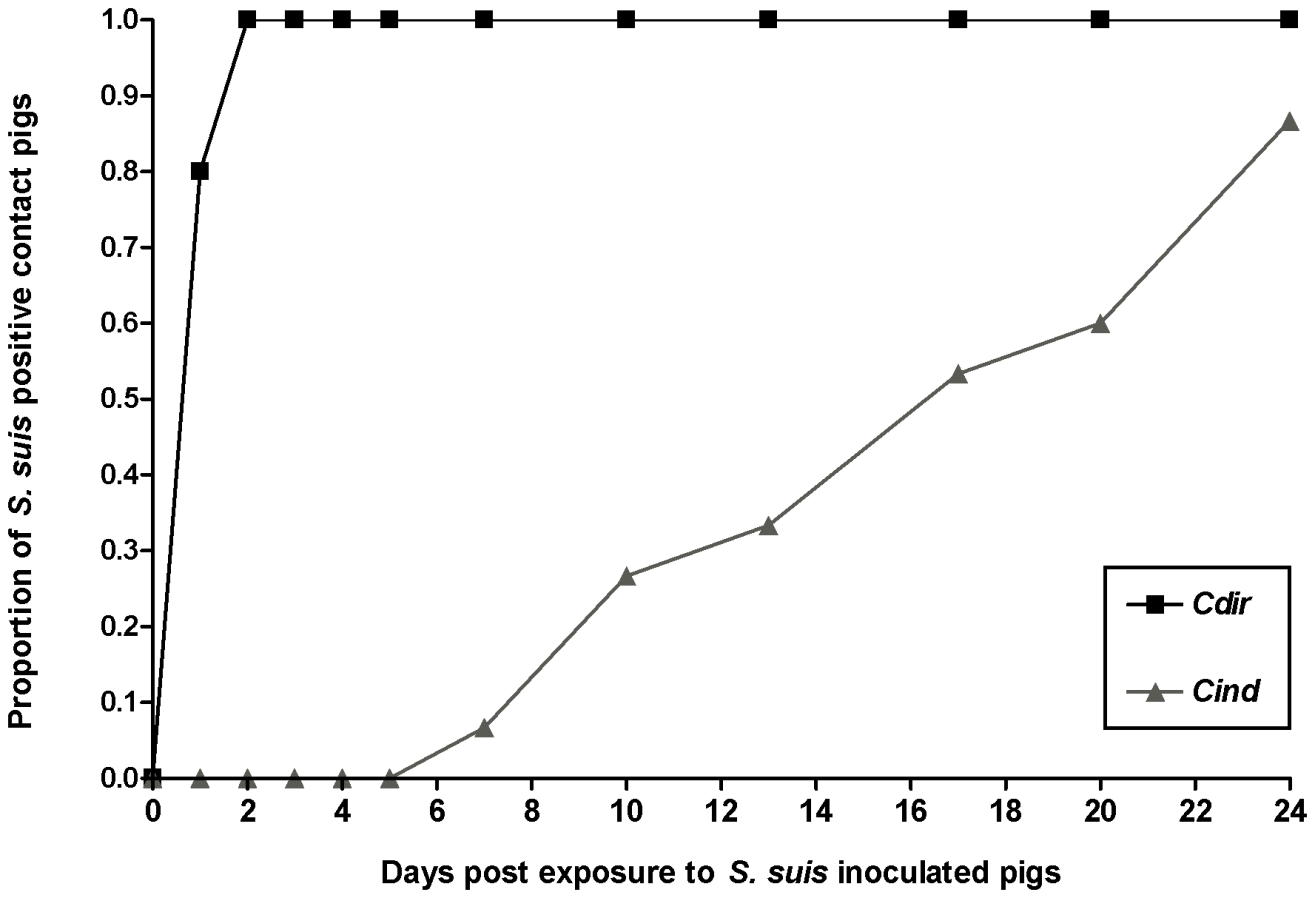
Transmission of *S.*suis to pigs with or without direct contact with infectious ones. Diagram showing the proportion of *S. suis* positive pigs over time for directly contact exposed pigs (*C_dir_*;N = 25) and pigs housed at a distance (*C_ind_*; N = 15) after *S. suis* serotype 9 inoculated pigs entered the stable compartments in the transmission experiments. Two *C_ind_* pigs remained negative.

No *S. suis* was detected in samples taken before inoculation. All inoculated pigs tested positive for *S. suis* at 2 DPI and all *C_dir_* pigs tested positive within two days after exposure. The first *C_ind_* pigs tested positive on day 7 post exposure and their number gradually increased to 13 (Figure 2). Once positive, pigs remained positive during the remaining experimental period.

**Figure 3:**
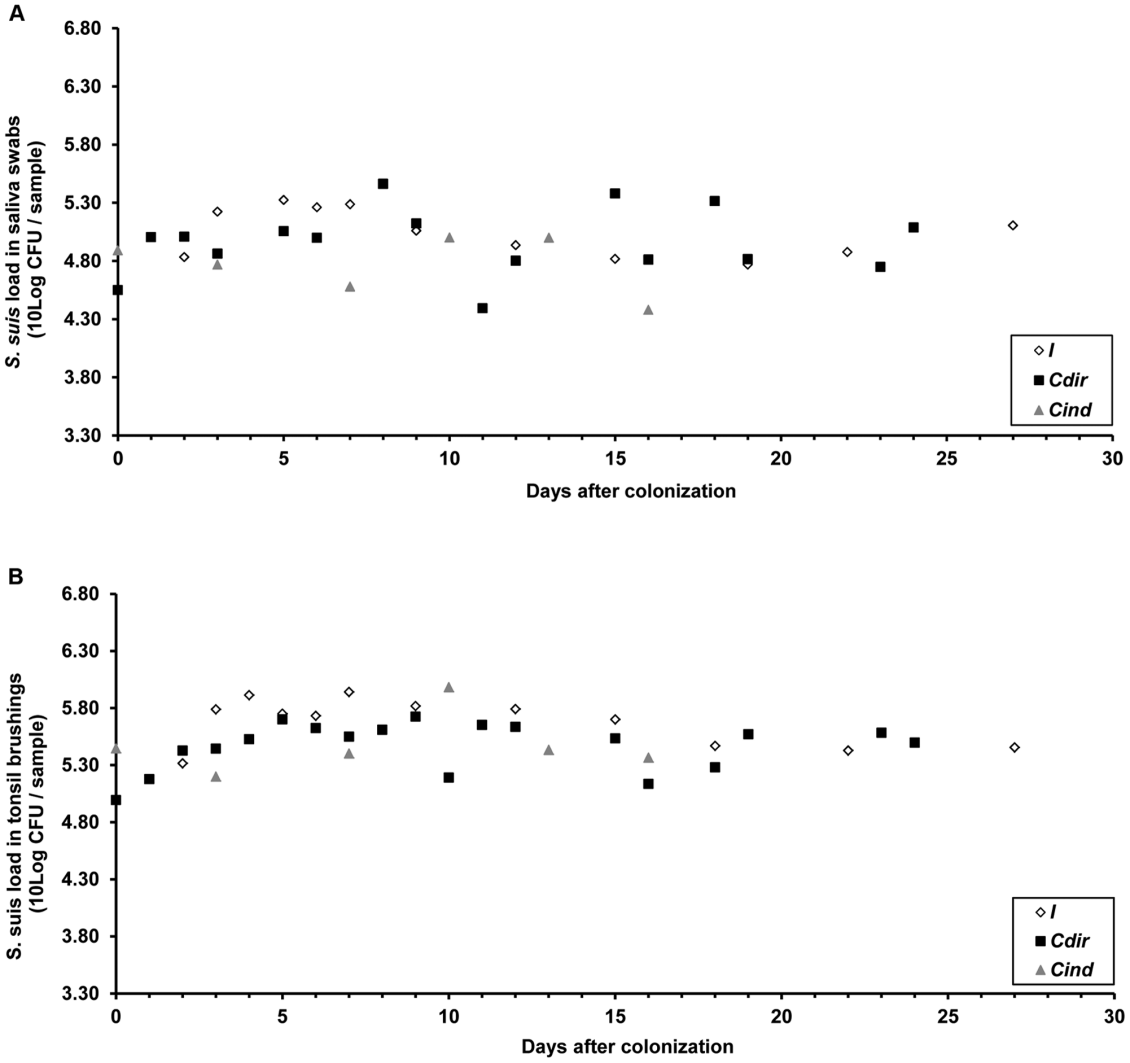
Mean *S.* suis loads in saliva and tonsil samples. Mean loads of *S. suis* serotype 9 colony forming units (CFU) in saliva (panel A) and tonsil brushing samples (panel B) shown separately for pigs inoculated with (*I*), directly (*C_dir_*) or indirectly contact exposed (*C_ind_*) to *S. suis*. Time is expressed as days after a pig was found positive firstly. As sampling was not conducted daily, and because of the delay in *S. suis* transmission to mainly *C_ind_* pigs the numbers and time points of observations differ between the groups. No significant differences were observed in mean levels between *C_dir_*, *C_ind_* and *I* pigs for the first 3 positive samples and for mean levels over the whole sampling period.

Mean levels of *S. suis* are shown in Figure 3. No differences were observed between the animal categories (*C_dir_*, *C_ind_* and *I)* in mean levels in tonsil and saliva samples (*P*-values are 0.25 and 0.12, respectively, for the first 3 positive samples and 0.19 and 0.66, respectively, for the whole sampling period). No differences between these categories were observed in the load of *S. suis* in their tonsil tissue samples (*P = *0.99). Based on these results, the assumption of homogeneity of infectivity was considered reasonable.

### Estimation of transmission rates

The rate for within-pen transmission *β_dir_* was estimated at 3.58 (95% CI: 2.29–5.60) new infections an animal will cause in a susceptible population, and for between-pen transmission *β’_ind_* was 0.001 (95% CI: 0.0006–0.0017) new infections an animal will cause each day in a susceptible population, for each day that it has already been infectious. Spatial separation reduced the transmission on average 36 times (range 20–895). Addition of cumulative *S. suis* levels did not change the fit of the model for estimating indirect transmission.

### Simulation study

**Figure 4:**
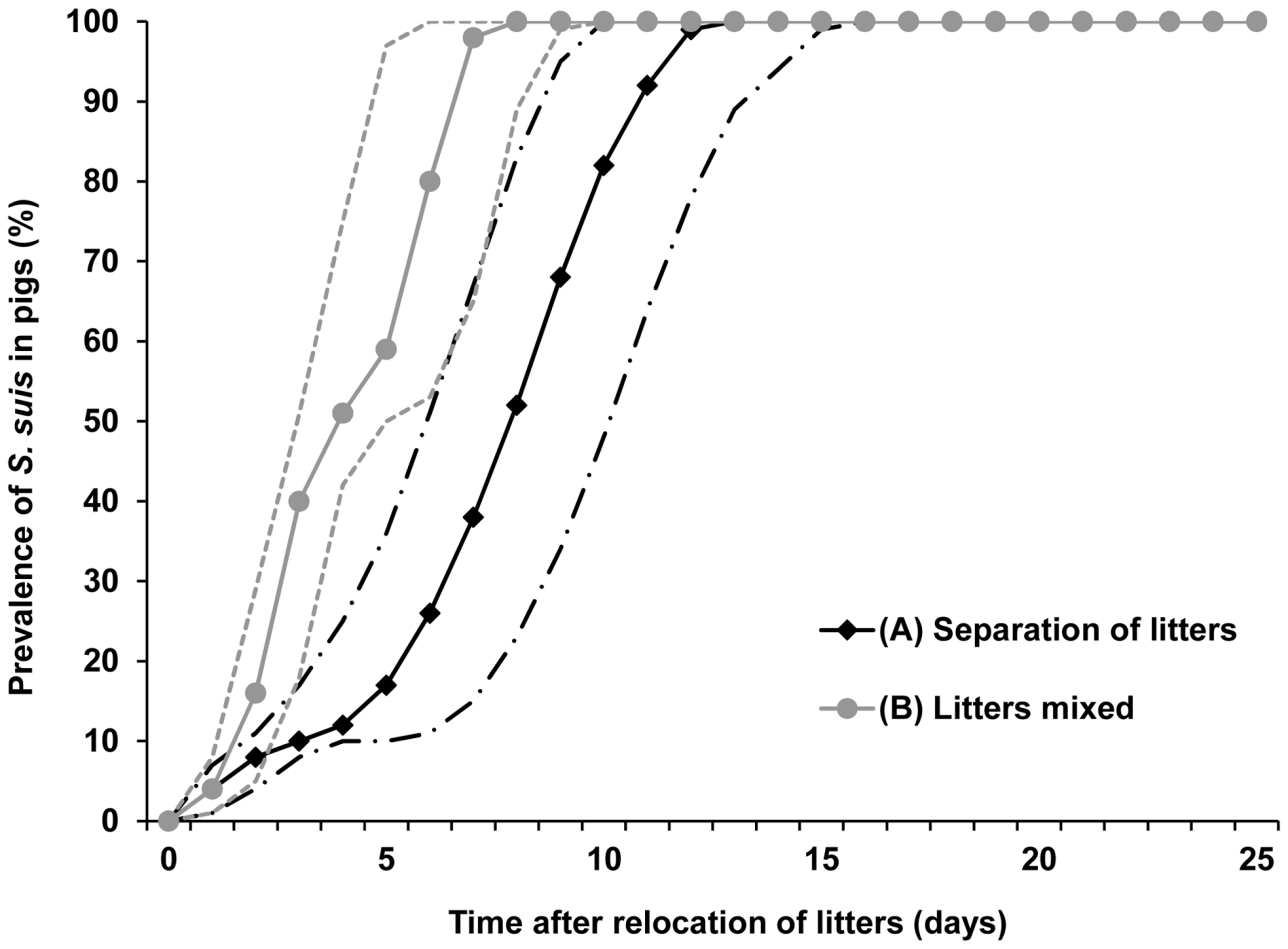
*S.* suis spread within a simulated hypothetical stable compartment under different weaning scenarios. Course of percentage of *S. suis* positive pigs over time after relocation of pigs at weaning for two simulated situations based on a hypothetical conventional pig farm setting. In scenario A, 10 litters of 10 pigs each were introduced in a stable compartment and kept spatially separated. In scenario B, 10 litters were mixed to form two groups of 50 pigs. In both scenarios it was assumed that that at relocation one of the 100 pigs was infected with *S. suis* and that direct contact between pigs in different pens was impossible. Per scenario 10.000 iterations were performed. The solid lines represent the median values, and the dashed lines the 2.5^th^ and 97.5^th^ percentiles of these simulations. The black lines represent scenario A, and the grey lines scenario B.

In both scenarios, all pigs were *S. suis* positive before 14 days (Figure 4). It was estimated that if *S. suis* free litters were separated from infected ones (scenario A), a prevalence of 50% or 90% would be reached at on average day 8 (2.5^th^–97.5^th^ percentile: 6–10 days) and day 11 (2.5^th^–97.5^th^ percentile: 9–13 days). When pigs were mixed (scenario B), this was reached after 4 (2.5^th^–97.5^th^ percentile: 3–5 days) and 7 days (2.5^th^–97.5^th^ percentile: 5–9 days), respectively.

### Clinical signs and mortality

Lameness was the only clinical sign observed in *C* pigs; it occurred in 4 out of 25 *C_dir_* and 3 out of 15 *C_ind_* pigs (*P* = 0.99). Signs were observed more often in *I* than in *C_dir_* (*P = *0.01) or *C_ind_* pigs (*P* < 0.001). Out of the 25 *I* pigs, in 20 pigs fever was recorded on one or more days, in 10 pigs lameness signs, in 3 pigs neurologic signs and 4 pigs died before the end of the experiment.

## Discussion

The objective of this study was to quantify the effect of separation of pigs on the transmission rate of *S. suis* serotype 9. All directly and indirectly exposed pigs became colonized after exposure to inoculated ones, but the rate at which it occurred was approximately 36 times lower for indirectly compared to directly exposed pigs. This finding suggests that this intervention measure could contribute to the reduction of *S. suis* spread in a farm. However, the simulation study using these parameters showed that the cumulative incidence in piglets did not differ between ‘separated’ and ‘mixed’ groups to a relevant level. This implies that it is unlikely that separation of pigs as simulated in this simulation exercise will contribute to reduction of the prevalence of *S. suis* serotype 9 infections in a farm compartment, also taking into account that on conventional farms less stringent hygienic measures are implemented than in this experiment.

Separation sharply reduced the transmission rate in the experiment, but had only a small effect on colonization time in a farm compartment. This counterintuitive finding is caused by the large number of pigs present in such a compartment. Although each of the indirectly exposed pigs only has a small probability of getting infected, the probability that at least one of them will contract the infection is considerable and, moreover, strongly increases as the number of infectious pigs in the ‘source’ pen increases. Once indirect transmission has taken place, direct transmission further increases the infectious load and consequently increases exposure of the remaining uninfected pigs and the probability of further indirect transmission.

In total 15 *C_ind_* pigs were colonized, most likely via contaminated dust particles, as has been described for *S. suis* serotype 2 [35], [48]. We consider other indirect routes of *S. suis* spread, e.g. via animal caretakers [20], [21], [49], less likely because of the stringent biosecurity measures that were applied.

A model assuming increasing infectivity fitted the data of indirect transmission better than a model assuming fixed infectivity. The increasing infectivity is likely to have occurred due to accumulation of *S. suis* in the environment [28], a phenomenon which has also been observed in studies with other pathogens [40], [50]–[55]. In studies with *Campylobacter jejuni* and *Escherichia coli* in broilers, for example, a similar pattern of increasing probability of infection was observed [40], [50]. The authors of these studies concluded that the model fitted the data better when assuming increasing infectivity [40], [50]. We also monitored the levels of the pathogen in the samples, as an association between these levels and an increasing infectivity could be suggested. The levels remained rather similar during the experiment, and addition of cumulative levels of *S. suis* in saliva or tonsil samples did not further improve the fit of the model.

Transmission of *S. suis* is not only dependent on the type of contacts between pigs, but also on the infectivity and susceptibility of individual pigs, which might be affected by (changes in) host behaviour. This may, for example, occur after mixing pigs from different litters leading to fighting [56]–[58], which would change the frequency and/or intensity of contacts between pigs [11], [12], [56]–[58]. Moreover, the stress induced by mixing [11], [12], [56]–[58] could further increase both susceptibility, and infectivity by increasing colonization and shedding of already infected hosts, as described for other bacterial pathogens [59]–[61]. If and to which extent the transmission changes after mixing is, however, not known. Moreover, even with adjustments the conclusions about the relevance of the effect of spatial separation would not change, as in the optimal ‘separation’ scenario the spread of *S. suis* serotype 9 was already so rapid that all pigs in a compartment were infected within about two weeks.

In the experiment stringent hygienic measures were applied between pens at compartment scale, and the same was assumed in the simulation study. In pig farms it is probably nearly impossible to perform such strict measures between pens in the same compartment, but they might be implemented at herd scale between compartments. As the rate of airborne spread is probably lower between compartments than between pens, spread of *S. suis* strains between different compartments, which are physically separated, might be reduced.

## Supporting Information

Table S1. Loads of S. suis in saliva, tonsillar brushing and tonsillar tissue samples, and clinical signs in individual pigs.
(DOCX)
