# Supplementary material for: Effect of Spatial Separation of Pigs on Spread of Streptococcus suis Serotype 9
Source: PLoS One. 2013 Apr 10;8(4):e61339. doi: 10.1371/journal.pone.0061339 (PMC3622602; doi:10.1371/journal.pone.0061339)
Supplement: Table S1. — Loads of S. suis in saliva, tonsillar brushing and tonsillar tissue samples, and clinical signs in individual pigs. (DOCX) [file pone.0061339.s001.docx]

***Supplementary Table S1. Loads of S. suis in saliva, tonsillar brushing and tonsillar tissue samples, and clinical signs in individual pigs.***

| **exp.**  **nr.** | **stable**  **unit** | **pig**  **nr.** | **vac.**  **status** | **exposure**  **status** | **day post exposure of contact pigs to inoculated pigs** | | | | | | | | | | | |  | **tonsil**  **tissue** |
| --- | --- | --- | --- | --- | --- | --- | --- | --- | --- | --- | --- | --- | --- | --- | --- | --- | --- | --- |
|  |  |  |  |  | **0** | **1** | **2** | **3** | **4** | **5** | **7** | **10** | **13** | **17** | **20** | **25** |  |  |
| I | A | 13 | nv | ino | **5.7/4.6^a^** | **6.2/6.6** | 5.9/6.3 | 5.4/6.1 | 4.9/5.4 | 6.1/6.0 | 5.3/5.2 | 4.8/5.2 | 4.9/3.6 | -/4.0 | 5.7/5.2 | **5.3/5.3** |  | **6.0** |
|  |  | 2 | nv | direct |  | **4.7/4.3** | **5.2/5.3** | 5.3/5.8 | 4.9/5.6 | 5.1/5.7 | 5.1/6.2 | 5.2/6.7 | 5.3/5.9 | 3.9/5.1 | 5.1/6.3 | **5.2/4.2** |  | **7.4** |
|  |  | 4 | v | ino | **5.5/6.3^a^** | **5.5/6.3** | 6.0/7.5 | 5.4/- | 5.2/5.6 | 4.8/6.1 | 5.3/6.3 | 4.1/5.5 | 5.3/5.4 | 3.6/3.6 | 4.9/5.3 | **5.2/5.5** |  | **-** |
|  |  | 8 | v | direct |  | **5.2/5.9** | **5.4/6.4** | 5.1/6.0 | 4.9/6.0 | 5.1/6.1 | 4.5/5.2 | 4.9/6.4 | 5.1/5.2 | -/3.9 | 5.2/5.6 | **4.6/5.1** |  | **6.5** |
|  |  | 3 | v | ino | **5.1/-**^a^ | **5.6/5.8** | 5.7/**6.3** | 5.7/6.3 | 5.8/5.8 | 5.3/6.4 | 4.9/6.1 | 4.7/5.4 | 5.0/5.3 | -/4.0 | 4.9/5.4 | **4.7/4.8** |  | **6.2** |
|  |  | 15 | v | direct |  | **5.8**/-* | **5.3/-** | **5.0/5.7** | 5.3/**5.5** | 5.4/- | 5.2/5.8 | 5.2/5.9 | 4.7/5.6 | -/4.0 | 4.9/4.7 | **4.6/5.3** |  | **7.3** |
|  |  | 7 | nv | indirect |  | -/- | -/- | -/- | -/- | -/- | -/- | -/- | -/- | -/- | -/- | **5.2/5.1** |  | - |
|  | B | 12 | nv | ino | **5.4/6.4^a^** | **4.4/5.6** | 5.5/5.1 | 4.5/5.6 | 5.3/6.0 | 6.0/6.8 | 4.7/6.1 | 5.4/6.3 | 5.0/5.5 | 5.6/6.6 | 5.4/5.6 | **5.4/5.5** |  | **6.6** |
|  |  | 6 | nv | direct |  | -/- | **5.1/5.9** | **4.4/5.0**^c^ | 5.2/6.0^c^ | 5.5/7.0^c^ | 5.3/6.0 | 5.1/6.4^c^ | 5.1/5.2^c^ | 5.8/6.7 | 5.7/5.4 | **4.0/5.7** |  | **6.9** |
|  |  | 10 | v | ino | **5.2/5.7** | **5.1/6.3** | 5.2/6.6 | 5.3/5.9 | 5.1/5.3 | 5.7/6.4 | 4.6/5.9 | 4.3/6.1 | 4.7/4.8 | 5.1/6.3 | 5.3/5.9 | **5.5/6.1** |  | **6.4** |
|  |  | 5 | v | direct |  | **5.8/5.3** | **6.3/5.4** | 5.1/5.3 | 4.7/5.3 | 4.9/6.0 | 5.0/5.4 | 5.3/6.3 | 4.8/5.6 | 5.3/5.8 | 5.8/5.7 | **5.2/5.7** |  | **6.5** |
|  |  | 1 | nv | indirect |  | -/- | -/- | -/- | -/- | -/- | -/- | **4.3/5.5** | **5.3/5.1** | 5.3/6.1 | 5.5/6.1 | **5.0/4.9** |  | **6.8** |
|  |  |  |  |  |  |  |  |  |  |  |  |  |  |  |  |  |  |  |
| II | A | 46 | nv | ino | **5.2/5.6^a^** | **5.4/5.9** | 5.8/5.5 | 6.0/5.9^a^ | 6.4/5.8^a^ | 6.3/6.4^d^ | 6.1/5.0^d^ | **† euth** |  |  |  |  |  | **6.9** |
|  |  | 33 | nv | direct |  | **4.1/5.5** | **4.1/5.4** | 5.0/4.7 | 4.7/5.1 | 4.6/6.0 | 4.7/4.8 | 4.9/5.5 | 3.6/5.9 | 5.4/6.4 | 5.1/5.6 | **5.4/5.6** |  | **6.5** |
|  |  | 42 | v | ino | **4.7/5.8** | **5.8/6.2^ab^** | 4.6/4.8^b^ | 5.3/5.5 | 4.6/- | 5.4/6.0 | 4.6/5.6 | 5.5/5.6 | 3.6/5.8 | 5.2/5.7 | 4.9/5.4 | **5.0/4.5** |  | **5.8** |
|  |  | 24 | v | direct |  | **5.3/4.8** | **4.5/4.7** | 4.6/5.4 | 4.4/5.6 | 5.2/6.3 | 5.2/6.0 | 5.7/6.2 | 5.4/6.7 | 5.2/6.5 | 5.4/5.6 | **4.5/5.3** |  | **6.8** |
|  |  | 34 | nv | ino | **- /** **6.0** | **5.1/5.4** | **5.6/**6.0**^a^** | 6.1/6.8^b^ | 5.6/5.9 | 4.1/5.7 | 5.0/5.5 | 5.5/5.8 | - /6.5 | 5.2/5.7 | 5.2/5.7 | **4.8/5.3** |  | **6.3** |
|  |  | 45 | nv | direct |  | -/- | **4.6/5.3** | **5.5/5.4** | 5.6/6.5 | 4.9/5.4 | 5.3/5.3 | 5.6/5.5 | -/6.6 | 5.4/5.5 | 4.5/5.8 | **4.7/5.7** |  | **6.0** |
|  |  | 41 | v | ino | **4.3/5.8^a^** | **5.5/6.2** | 4.3/5.5 | 4.8/6.1 | 5.0/6.1 | 5.4/6.5 | 5.2/5.7 | 5.2/5.4 | 3.6/5.8 | 4.4/5.6 | 4.3/5.9 | **4.4/5.2** |  | **5.8** |
|  |  | 17 | v | direct |  | **5.0/5.3** | -/- | **4.9/5.5** | 4.4/5.2 | 5.9/5.9 | 5.4/5.9 | 5.7/5.9 | 4.5/6.3 | 4.5/5.7 | 4.7/4.7 | **4.6/5.8** |  | **5.9** |
|  |  | 19 | nv | indirect |  | -/- | -/- | -/- | -/- | -/- | 5.3/5.4 | 4.6/5.7 | 4.6/6.1 | 4.9/6.1 | 5.2/5.6 | **4.4/5.4** |  | **6.5** |
|  |  | 35 | nv | indirect |  | -/- | -/- | -/- | -/- | -/- | -/- | -/- | -/- | -/- | -/- | **5.1/5.6** |  | **5.7** |
|  | B | 32 | nv | ino | **5.2/5.8^ab^** | **4.4/3.6** | 4.9/6.2 | 6.2/ 5.7 | 5.9/ 6.2 | 4.8/5.1^b^ | 5.1/6.0^b^ | 4.7/6.2 | 5.2/5.4 | 5.5/5.6 | 5.0/6.1 | **6.2/ 6.3** |  | **6.1** |
|  |  | 39 | nv | direct |  | -/- | **5.3/5.5** | **6.2/5.3** | 5.8/5.8^c^ | 4.8/5.5 | 5.0/5.9 | 5.3/5.7 | - /5.5 | 5.4/5.7 | 5.6/5.9 | **5.0/5.7** |  | **6.1** |
|  |  | 18 | v | ino | **4.6/5.7** | **5.0/5.3** | 5.4/5.4 | **†** |  |  |  |  |  |  |  |  |  | **6.9** |
|  |  | 22 | v | direct |  | **4.1/-** | **4.4/4.8** | 4.4/**5.0** | 4.6/5.1 | 5.0/4.9 | 4.8/4.7 | -/4.8 | 4.2/5.0 | 5.1/3.8 | 4.8/5.6 | **3.8/5.4** |  | **6.1** |
|  |  | 20 | nv | ino | **5.3/5.0^a^** | **4.9/4.8** | 4.9/5.9^a^ | 6.3/6.0 | 5.3/5.7 | 4.8/5.1 | 5.0/5.8 | 5.5/5.8 | 4.8/5.6 | 5.4/6.1 | 5.2/5.9 | **5.2/5.3** |  | **6.9** |
|  |  | 30 | nv | direct |  | **6.6/5.5** | **5.1/5.5** | 5.5/5.5 | 5.3/5.4 | 4.7/5.7 | 5.4/5.9 | 4.6/5.6 | 4.1/6.1 | 5.2/5.2 | 4.4/5.8 | **5.0/5.5** |  | **6.6** |
|  |  | 25 | v | ino | **4.4/5.6** | **-/5.7** | **5.0**/5.9 | 5.6/5.7 | 5.9/6.2 | 5.3/5.6 | 5.5/6.2 | 5.7/5.8 | 4.6/7.8 | 5.4/5.5 | 4.6/5.6 | **4.5/5.7** |  | **5.7** |
|  |  | 16 | v | direct |  | **-/4.7** | **5.5/5.5** | **6.2**/5.9 | 6.1/5.9 | 5.4/5.6 | 5.3/5.9 | 5.8/5.2 | 4.9/5.5 | 5.5/- | 4.8/5.8 | **5.4/6.1** |  | **6.3** |
|  |  | 21 | nv | indirect |  | -/- | -/- | -/- | -/- | -/- | -/- | -/- | -/- ^c^ | **5.1/5.4** | **4.8/5.9** | **4.9/6.4** |  | - |
|  |  | 29 | nv | indirect |  | -/- | -/- | -/- | -/- | -/- | -/- | -/- | -/- | -/- | -/- | **4.3/7.1** |  | **6.4** |
| **exp.**  **nr.** | **stable**  **unit** | **pig**  **nr.** | **vac.**  **status** | **exposure**  **status** | **day post exposure of contact pigs to inoculated pigs** | | | | | | | | | | | |  | **tonsil**  **tissue** |
|  |  |  |  |  | **0** | **1** | **2** | **3** | **4** | **5** | **7** | **10** | **13** | **17** | **20** | **25** |  |  |
| II | C | 40^e^ | v | ino | **4.3/5.3^b^** | **5.1/6.5** | 5.4/6.3^b^ | 6.0/6.6^b^ | 5.5/6.2^b^ | 4.4/6.3^b^ | 5.0/6.1 | 5.1/5.5 | 4.7/6.4 | 5.2/6.0 | 5.6/- | **4.6/6.5** |  | **6.3** |
|  |  | 27 | v | direct |  | **4.8/6.5** | **6.1/4.8** | 5.7/5.7 | 5.4/5.3 | 5.2/5.4 | 5.7/5.6 | 5.7/5.5 | 4.6/5.8 | 4.0/5.7 | 5.4/5.7 | **5.0/5.9** |  | **5.9** |
|  |  | 23 | v | ino | **5.4/5.5^ab^** | **5.6/6.6^ad^** | 5.5/6.6^d^ | 5.7/5.9^c^ | 5.4/6.3^c^ | 5.4/6.7 | 5.2/6.2 | 5.4/5.9 | 5.9/6.7^ac^ | 6.0/7.3^b^ | 5.3/5.9^d^ | **5.2/5.1^d^** |  | **6.6** |
|  |  | 44 | v | direct |  | **-/4.3** | **4.5/4.3** | **4.3**/4.7 | -/5.4 | 4.9/6.1 | 5.1/5.5 | 6.4/6.4 | 4.6/4.2 | 5.2/6.3 | 5.3/5.7 | **5.6/5.4** |  | **6.7** |
|  |  | 43 | nv | indirect |  | -/- | -/- | -/- | -/- | -/- | -/- | -/- | -/- | -/- | **5.3/6.3** | **5.5/-** |  | **7.0** |
|  |  | 36 | nv | indirect |  | -/- | -/- | -/- | -/- | -/- | -/- | -/- | **-/3.8** | **4.8/5.8** | **5.7**/5.8 | **5.3/6.5** |  | **6.8** |
| III | A | 65 | nv | ino | **4.3/5.5** | **4.6/5.3** | 5.1/5.7 | 5.1/5.6 | 5.2/5.5 | 4.8/5.6 | 4.9/5.4 | 4.3/5.6 | 5.3/5.4 | 4.4/5.6 | 3.9/5.6 | **5.5/5.5** |  | **6.4** |
|  |  | 69 | nv | direct |  | **4.1/4.5** | **4.4/5.5** | 5.0/5.2 | 5.2/5.4 | 4.2/5.3 | 4.5/5.6 | 5.5/5.7 | 4.6/5.7 | 5.3/5.6 | 4.1/5.7 | **5.2/5.6** |  | **6.8** |
|  |  | 53 | v | ino | **5.5/5.0^ad^** | **6.4/6.6^ad^** | 5.5/6.0^ad^ | 5.3/5.3^ad^ | 5.1/5.7^ad^ | **† euth** |  |  |  |  |  |  |  | **6.1** |
|  |  | 55 | v | direct |  | -/- | **-/5.1** | **4.5/4.7** | **4.6**/4.9 | 4.5/5.5 | 5.0/5.6 | 5.8/5.7 | 5.7/5.5 | 5.5/5.9 | 5.9/4.4 | **5.2/5.5**^c^ |  | **6.0** |
|  |  | 77 | nv | ino | **4.3/ -** | **5.4/5.9** | 5.5**/5.9** | 4.6/5.5 | 5.6/5.7 | 4.9/5.3 | 4.6/5.9 | 4.5/5.5 | 5.3/5.8 | 5.4/ - | 4.3/5.3 | **5.2/5.9** |  | **5.9** |
|  |  | 60 | nv | direct |  | **5.1/5.6** | **5.4/5.6** | 5.2/5.7 | 4.7/5.5 | 5.1/5.7 | 4.5/5.4 | 4.7/5.6 | 5.4/5.5 | 5.2/5.4 | 4.6/5.9 | **6.1/4.6**^c^ |  | **6.8** |
|  |  | 52 | v | ino | **4.4/4.9^a^** | **5.5/5.4** | 6.5/6.1^ad^ | 6.3/6.3^ad^ | **† euth** |  |  |  |  |  |  |  |  | **6.4** |
|  |  | 58 | v | direct |  | **3.9/5.5** | **5.2/5.5** | 4.8/5.8 | 4.5/5.9 | 4.9/5.2 | 4.7/5.1 | 5.3/5.5 | 4.6/5.5 | 4.8/5.4 | -/5.1 | **5.2/5.1** |  | **6.3** |
|  |  | 76 | nv | indirect |  | -/- | -/- | -/- | -/- | -/- | -/- | **5.0/5.4** | **4.3/5.4** | 5.4/5.6 | 4.6/5.4 | **4.6/5.6**^c^ |  | **7.0** |
|  |  | 68 | nv | indirect |  | -/- | -/- | -/- | -/- | -/- | -/- | **4.6/5.1** | **4.6/4.9** | -/- | 5.0/5.8 | **5.2/5.6** |  | **6.7** |
|  | B | 61 | nv | ino | **4.5/5.3^ab^** | **5.9/5.4^b^** | 5.4/5.7^c^ | 5.0/5.2^ac^ | 5.1/5.1^ac^ | 4.7/5.7^b^ | 5.3/5.0 | 4.3/5.8 | 5.1/5.3 | 4.6/5.8 | 4.1/4.9 | **5.6/5.3** |  | **6.4** |
|  |  | 71 | nv | direct |  | **4.8/4.5** | **4.8/5.3** | 4.7/4.6 | 4.8/5.0 | 4.7/5.6 | 4.6/5.7 | 4.4/5.2 | 5.7/5.6 | 4.7/5.5 | 4.1/5.5 | **4.9/4.4** |  | **5.7** |
|  |  | 75 | nv | ino | **4.6/5.5** | **5.1/5.7** | 4.8/5.5 | 4.9/5.5^b^ | 3.8/5.7 | 5.6/5.3 | 5.0/5.0^b^ | 5.6/6.2 | 4.9/5.5 | 5.3/5.8 | 4.7/5.8 | **5.8/4.1^b^** |  | **7.2** |
|  |  | 72 | nv | direct |  | -/- | **5.4/6.3** | **5.3/5.5** | 4.3/5.4 | 5.1/5.9 | 4.8/6.5 | 5.4/5.9 | 4.6/5.6 | 4.7/5.4 | 4.9/5.4 | **4.7/5.5** |  | **6.2** |
|  |  | 67 | nv | ino | **6.2/6.3^b^** | **5.5/6.0** | 5.2/6.2^c^ | 5.0/5.3^c^ | 4.6/6.8^b^ | 5.5/6.0^b^ | 5.1/6.4 | 5.3/5.9 | 5.1/5.6 | 3.8/5.3 | 5.4/5.5 | **4.5/5.1** |  | **6.0** |
|  |  | 66 | nv | direct |  | **-3.8** | **5.0/5.2** | **4.1/**4.4 | 4.7/5.5 | 5.1/5.4 | 5.0/5.4 | 4.8/5.5 | 4.8/5.2 | 5.5/5.4 | -/5.2 | **5.3/5.6** |  | **6.5** |
|  |  | 54 | v | ino | **5.2/-** | **5.9/5.9** | 5.1/**5.7** | 5.7/5.1^c^ | 4.8/5.5 | 5.2/5.9 | 5.3/6.4 | 4.6/5.8^c^ | 5.0/6.2 | 4.7/5.8^b^ | 4.4/5.6^b^ | **5.0/5.3** |  | **6.3** |
|  |  | 56 | v | direct |  | **4.9/5.7** | **4.4/5.8** | 5.3/5.5 | 4.7/5.7 | 5.1/5.6 | 5.2/6.2 | 5.3/5.9 | 4.7/5.6 | 4.4/5.6 | 4.8/5.3 | **5.5/5.1** |  | **5.7** |
|  |  | 64 | nv | indirect |  | -/- | -/- | -/- | -/- | -/- | -/- | -/- | -/- | **5.5/5.2** | **4.6/5.6** | **4.6/5.2** |  | **6.6** |
|  |  | 70 | nv | indirect |  | -/- | -/- | -/- | -/- | -/- | -/- | -/- | -/- | **3.9/5.2** | **3.6/5.2** | **-/4.7** |  | **6.8** |
|  | C | 59 | nv | ino | **4.7/5.7** | **4.4/6.1** | 4.9/5.6 | 5.4/5.6 | 5.3/5.6^b^ | 5.9/5.9 | 4.9/6.0 | 5.0/6.2 | 5.2/5.5 | 4.7/5.6 | 4.0/5.8 | **4.9/5.7^c^** |  | **6.6** |
|  |  | 73 | nv | direct |  | **4.0/5.0** | **5.3/5.6** | 4.5/5.5 | 5.6/5.5 | 5.8/5.6 | 4.9/5.7 | 4.7/5.8 | 5.2/5.9 | 5.2/5.9 | 5.6/5.8 | **5.2/5.5** |  | **6.2** |
|  |  | 51 | v | ino | **-/5.6** | **5.0/5.7** | **5.3**/5.9^c^ | 4.8/5.6^ac^ | 5.4/6.3 | 5.6/6.0 | 4.9/5.9 | 4.0/6.0 | 4.8/5.7 | 4.1/5.9 | 5.1/4.3 | **4.9/6.5** |  | **6.7** |
|  |  | 57 | v | direct |  | **5.0/5.2** | **5.1/5.6** | 4.9/4.9 | 4.7/5.0 | 4.9/4.8 | 5.1/5.7 | 5.0/6.1 | 5.2/5.4 | 5.1/4.8 | 5.5/5.6 | **5.6/5.6** |  | **6.2** |
|  |  | 63 | nv | indirect |  | -/- | -/- | -/- | -/- | -/- | -/- | -/- | -/- | -/- | -/- | -/- ^c^ |  | - |
|  |  | 74 | nv | indirect |  | -/- | -/- | -/- | -/- | -/- | -/- | -/- | -/- | -/- | -/- | **5.1/5.6** |  | - |
|  |  | 62 | nv | indirect |  |  |  |  |  |  |  |  |  |  |  | -/- |  | - |

*(Continuation of table on previous page)*

*(Continuation of table on previous page)*

***Captions:***

**exp.**= experiment; **ino** = inoculated pigs; **direct** = housed in one box with an inoculated pig; **indirect**= individually housed at a distance of ± 1 m of infectious pairs; **†** = found dead; **† euth** = euthanized for animal welfare reasons. The pairwise housed pigs are shown together: the direct contact pig directly below the inoculated pig.

***** ‘**…../…..**’ = ***saliva / tonsil***, e.g. ***‘5.8 /-***’ means that no *S. suis* serotype 9 was isolated from tonsil brushing, while the saliva sample was positive and contained a load of 5.8 ^10^LogCFU; a bold number indicates that isolates from this sample were tested in the *cps9H*-PCR-test, and found positive.

****** These values represent the loads of *S. suis* in ^10^LogCFU per gram of tonsillar tissue.

**^a^** body temperature ≥ 40°C.

**^b^** lameness, degree 1 (i.e. avoiding movement of leg(s)).

**^c^** lameness degree 2 (i.e. avoiding bearing weight on leg(s)).

**^d^** neurologic signs.

**^e^** pig had mild locomotory problems before inoculation
